# Propensity score-matched analysis comparing dose-escalated intensity-modulated radiation therapy versus external beam radiation therapy plus high-dose-rate brachytherapy for localized prostate cancer

**DOI:** 10.1007/s00066-022-01953-y

**Published:** 2022-05-12

**Authors:** Jörg Tamihardja, Ingulf Lawrenz, Paul Lutyj, Stefan Weick, Matthias Guckenberger, Bülent Polat, Michael Flentje

**Affiliations:** 1 Department of Radiation Oncology, University of Wuerzburg, Josef-Schneider-Str. 11, 97080 Würzburg, Germany; 2 Department of Radiation Oncology, University Hospital Zurich, University of Zurich, Zurich, Switzerland

**Keywords:** Long-term outcome, Dose escalation, High-dose-rate brachytherapy boost, Propensity score matching, Toxicity

## Abstract

**Purpose:**

Dose-escalated external beam radiation therapy (EBRT) and EBRT + high-dose-rate brachytherapy (HDR-BT) boost are guideline-recommended treatment options for localized prostate cancer. The purpose of this study was to compare long-term outcome and toxicity of dose-escalated EBRT versus EBRT + HDR-BT boost.

**Methods:**

From 2002 to 2019, 744 consecutive patients received either EBRT or EBRT + HDR-BT boost, of whom 516 patients were propensity score matched. Median follow-up was 95.3 months. Cone beam CT image-guided EBRT consisted of 33 fractions of intensity-modulated radiation therapy with simultaneous integrated boost up to 76.23 Gy (D_Mean_). Combined treatment was delivered as 46 Gy (D_Mean_) EBRT, followed by two fractions HDR-BT boost with 9 Gy (D_90%_). Propensity score matching was applied before analysis of the primary endpoint, estimated 10-year biochemical relapse-free survival (bRFS), and the secondary endpoints metastasis-free survival (MFS) and overall survival (OS). Prognostic parameters were analyzed by Cox proportional hazard modelling. Genitourinary (GU)/gastrointestinal (GI) toxicity evaluation used the Common Toxicity Criteria for Adverse Events (v5.0).

**Results:**

The estimated 10-year bRFS was 82.0% vs. 76.4% (*p* = 0.075) for EBRT alone versus combined treatment, respectively. The estimated 10-year MFS was 82.9% vs. 87.0% (*p* = 0.195) and the 10-year OS was 65.7% vs. 68.9% (*p* = 0.303), respectively. Cumulative 5‑year late GU ≥ grade 2 toxicities were seen in 23.6% vs. 19.2% (*p* = 0.086) and 5‑year late GI ≥ grade 2 toxicities in 11.1% vs. 5.0% of the patients (*p* = 0.002); cumulative 5‑year late grade 3 GU toxicity occurred in 4.2% vs. 3.6% (*p* = 0.401) and GI toxicity in 1.0% vs. 0.3% (*p* = 0.249), respectively.

**Conclusion:**

Both treatment groups showed excellent long-term outcomes with low rates of severe toxicity.

## Introduction

External beam radiation therapy (EBRT) and EBRT combined with high-dose-rate brachytherapy (HDR-BT) boost are well-established options for treating localized prostate cancer. Both radiation therapy modalities are widely practiced, but the question arises of which modality offers the best oncologic outcome while minimizing toxicity. Although HDR-BT offers excellent dose conformity, EBRT may be beneficial for ablating periprostatic disease extension in high-risk cancer [1]. Several randomized controlled trials showed an increase in biochemical control by dose escalation, which Hoskin et al. was able to demonstrate for the addition of HDR-BT boost to EBRT [2–7]. While dose-escalated EBRT + HDR-BT boost has proven to be superior to EBRT with an EQD2 of 66–74 Gy, data on the comparison of dose-escalated treatment modalities above 80 Gy remain scarce [7]. As dose escalation above 80 Gy remains controversial and has yet to show an improvement of clinically important outcome parameters, the present study aims at improving the evidence base for dose escalation beyond 80 Gy [8, 9].

In the absence of randomized controlled trials for the comparison of dose-escalated EBRT above 80 Gy versus EBRT + HDR-BT boost, we performed a propensity score matching-based single-center analysis to address this question. Specifically, we compared the long-term biochemical relapse-free survival (bRFS), metastasis-free survival (MFS), overall survival (OS), and the long-term side effects of dose-escalated EBRT with an equivalent dose in 2 Gy fractions (EQD2) of 83 Gy (α/β 1.5 Gy) versus combined dose-escalated EBRT + HDR-BT boost with an EQD2 of 100 Gy for localized prostate cancer.

## Materials and methods

### Study design and participants

This retrospective single-center analysis is based on 744 consecutive patients treated between 2002 and 2019 with either EBRT (*n* = 406) or EBRT + HDR-BT boost (*n* = 338) for localized prostate cancer. All patients had pathologically confirmed prostate cancer. Stratification was conducted according to the risk group classification of D’Amico et al. [10]. Additive androgen deprivation therapy was prescribed at the discretion of the treating urologist and recommended for patients with intermediate-risk (6 months) and high-risk disease (24–36 months).

### Treatment

The treatment procedures have been described in detail before and will be summarized briefly in the following [11, 12]. For the EBRT cohort, radiation therapy was delivered with intensity-modulated radiation therapy (IMRT) or volumetric modulated arc therapy (VMAT) in 33 fractions with simultaneous integrated boost and two dose levels of 1.82 Gy and 2.31 Gy per fraction, resulting in a prescribed planning target volume (PTV) dose of 60.06 Gy (D_95_) and a PTV_Boost_ mean dose of 76.23 Gy. Concerning contouring, a clinical target volume (CTV), CTV_P−SV_, was generated which consisted of the prostate and the proximal seminal vesicles, whereas the CTV_P+SV_ included the prostate and the whole seminal vesicles. The PTV_Boost_ was defined by placing a 5-mm margin around CTV_P−SV_ with avoidance of the rectum. The PTV was created with a 10-mm margin around CTV_P+SV_ in all but the dorsal direction, where a 7-mm margin was applied.

For the combined treatment cohort, EBRT was delivered with 3D-conformal radiation therapy, IMRT, or VMAT in 23 fractions with 2 Gy per fraction, resulting in a prescribed PTV dose of 46 Gy (D_Mean_). A CTV was generated consisting of the prostate and the seminal vesicles. The PTV was created by a 10-mm margin around the CTV in all but the dorsal direction, where a 7 mm margin was used. Approximately 2 weeks after completion of EBRT, two HDR-BT boost fractions were performed with a 14-day interval between the two applications. The HDR-BT boost PTV was defined as the entire prostate without the seminal vesicles and additional margin. The prescription dose for the PTV was 9 Gy (D_90%_) per fraction. Pinnacle^3^ (Philips Radiation Oncology Systems, Fitchburg, WI, USA) was used for EBRT treatment planning for both treatment cohorts.

### Outcomes

Biochemical failure was defined according to the Phoenix definition as nadir plus a ≥ 2-ng/ml increase in prostate-specific antigen (PSA). Biochemical relapse-free survival, defined as the time between the conclusion of radiation therapy treatment and the date of biochemical failure, was the primary reported endpoint of this retrospective study. Secondary endpoints were metastasis-free survival, overall survival, and the 5‑year cumulative incidence of gastrointestinal and genitourinary toxicity. Metastasis-free survival was defined as the time between the end of radiation therapy and the date of occurrence of distant metastasis diagnosed by imaging. The time between the end of treatment and the date of death from any cause was defined as overall survival. Patients alive or lost to follow-up at the time of analysis were censored at the date of last contact. Follow-up was defined as the time between the date of the end of radiation therapy and the date of last news. Assessment of physician-recorded toxicity during radiation therapy was performed at baseline, at the end of treatment, 6 weeks after treatment, and at 6‑month intervals thereafter. After 2 years, follow-up was changed to longer periods with annual examination. Gastrointestinal (GI) and genitourinary (GU) toxicity were scored using the Common Terminology Criteria for Adverse Events (CTCAE) v5.0. We assessed side effects systematically according to CTCAE v5.0 during follow-ups as well as by reviewing the patient files. Acute toxicity was defined as occurring between the start of radiation therapy and until 3 months after the end of radiation therapy. All subsequent follow-ups were included in the late toxicity evaluation.

### Statistical analysis

A propensity score-matched analysis with a 1:1 ratio and a caliper of 0.1 was conducted to balance the significantly different baseline patient and histopathologic characteristics between the two treatment groups in order to minimize their effect on the oncologic outcome. A logistic regression model was used to calculate the propensity score, which included the following variables: patient age at the start of radiation therapy, Gleason score, initial PSA value, stage, use of ADT, and risk group according to D’Amico. All survival curves were estimated using the Kaplan–Meier method with the date of the end of radiation therapy as baseline time. For categorical data χ^2^ tests and for continuous variables Mann–Whitney U tests were used to compare baseline characteristics for unmatched and matched datasets. The log-rank test was used to compare survival curves. On the matched dataset, multivariable Cox proportional hazard models including hazard ratios (HR) with 95% confidence intervals (CI) were run using propensity score matching variables and type of radiation therapy treatment to uncover prognostic factors for oncologic outcome. For all statistical analyses, the R software environment for statistical computing and graphics (version 4.1; The R Foundation for Statistical Computing, Vienna, Austria) was used. All tests were two sided with statistical significance indicated by *p* < 0.05.

## Results

Before propensity score matching, significant differences in patient age at the start of radiation therapy, initial PSA, Gleason score, stage, and D’Amico risk group were present. Patients in the combined treatment group had increased unfavorable disease characteristics (higher initial PSA, higher Gleason score, higher stage), and belonged more often to the high-risk group according to D’Amico. Irradiation of the pelvic lymph nodes occurred more often in the combined treatment group for the unmatched dataset. In the matched dataset, the pelvic lymph nodes were irradiated in 32.9% of all patients in the EBRT-only group and in 30.6% in the EBRT + HDR-BT group (*p* = 0.612). Additive ADT was given in 53.5% versus 51.9% (*p* = 0.724) with a median duration (interquartile range) of 609 days (348–1013 days) versus 548 days (206–777 days) for the EBRT-only group versus the combined treatment group, respectively (*p* = 0.089). All clinical, pathological, and treatment-related characteristics before and after propensity score matching are shown in Table 1.

**Table 1:** Baseline characteristics for patients who underwent external beam radiotherapy versus those who received external beam radiotherapy plus high-dose-rate brachytherapy boost before and after propensity score matching

Characteristic	Unmatched (complete) dataset			Matched (1:1) dataset		
	EBRT (*N* = 406)	EBRT + HDR-BT (*N* = 338)	*p*-value*	EBRT (*N* = 258)	EBRT + HDR-BT (*N* = 258)	*p*-value^a^
*Follow-up (months)*	68.3 (49.3, 104.5)	101.8 (65.7, 143.0)	< 0.001	75.9 (53.8, 117.2)	110.2 (67.5, 150.7)	< 0.001
*KPS (%)*	90 (90, 100)	90 (90, 100)	< 0.001	90 (80, 100)	90 (90, 100)	< 0.001
*Age at RT (years)*	73.7 (68.6, 76.9)	69.0 (64.3, 73.3)	0.001	72.3 (66.8, 76.4)	71.0 (66.0, 74.5)	0.003
*PSA at diagnosis (ng/mL)*	8.3 (5.4, 16.4)	10.1 (6.5, 20.4)	0.003	10.2 (6.3, 21.6)	10.5 (6.4, 21.5)	0.860
*PSA at diagnosis, n (%)*	–	–	0.010	–	–	0.779
< 10 ng/mL	242 (59.6)	164 (48.5)	–	126 (48.8)	121 (46.9)	–
10–20 ng/mL	81 (20.0)	88 (26.0)	–	63 (24.4%)	70 (27.1)	–
> 20 ng/mL	83 (20.4)	86 (25.4)	–	69 (26.7%)	67 (26.0)	–
*Gleason score, n (%)*	–	–	< 0.001	–	–	0.197
≤ 6	133 (32.8)	63 (18.6)	–	61 (23.6)	55 (21.3)	–
7	170 (41.9)	173 (51.2)	–	120 (46.5)	140 (54.3)	–
≥ 8	99 (24.4)	100 (29.6)	–	77 (29.8)	63 (24.4)	–
Missing, *n* (%)	4 (1.0)	2 (0.6)	–	–	–	–
*Stage, n (%)*	**–**	**–**	< 0.001	**–**	**–**	0.144
≤ T2a	316 (77.8)	206 (60.9)	–	175 (67.8)	165 (64.0)	–
T2b	7 (1.7)	13 (3.8)	–	5 (1.9)	13 (5.0)	–
≥ T2c	83 (20.4)	119 (35.2)	–	78 (30.2)	80 (31.0)	–
*D’Amico risk class, n (%)*	–	–	< 0.001	–	–	0.073
Low	88 (21.7)	15 (4.4)	–	23 (8.9)	13 (5.0)	–
Intermediate	142 (35.0)	115 (34.0)	–	85 (32.9)	105 (40.7)	–
High	176 (43.3)	208 (61.5)	–	150 (58.1)	140 (54.3)	–
*Lymph node RT, n (%)*	–	–	0.004	–	–	0.612
Yes	101 (24.9)	116 (34.3)	–	85 (32.9)	79 (30.6)	–
No	305 (75.1)	222 (65.7)	–	173 (67.1)	177 (68.6)	–
*ADT, n (%)*	–	–	0.067	–	–	0.724
Yes	184 (45.3)	176 (52.1)	–	138 (53.5)	134 (51.9)	–
No	222 (54.7)	162 (47.9)	–	120 (46.5)	124 (48.1)	–
*ADT duration (days)*	637 (352, 1022)	594 (274, 823)	0.129	609 (348, 1013)	548 (206, 777)	0.089
*MRI staging, n (%)*						
Yes	253 (62.3)	79 (23.4)	< 0.001	154 (59.7)	49 (19.0)	< 0.001
No	153 (37.7)	259 (76.6)	–	104 (40.3)	209 (81.0)	–
*PET-CT after RT, n (%)*	–	–	0.074	–	–	0.880
Yes	30 (7.4)	38 (11.2)	–	23 (8.9)	25 (9.7)	–
No	376 (92.6)	300 (88.8)	–	235 (91.1)	333 (90.3)	–

Estimates were given as median (quartile 1, quartile 3) or frequency (percentage)

*ADT* androgen deprivation therapy, *EBRT* external beam radiation therapy, *HDR-BT* high-dose-rate brachytherapy, *KPS* Karnofsky performance status, *PET-CT* positron-emission tomography computed tomography, *PSA* prostate-specific antigen, *RT* radiation therapy

^a^*p*-values were calculated using Mann–Whitney U test for continuous and χ^2^ test for categorical variables

**Fig. 1:**
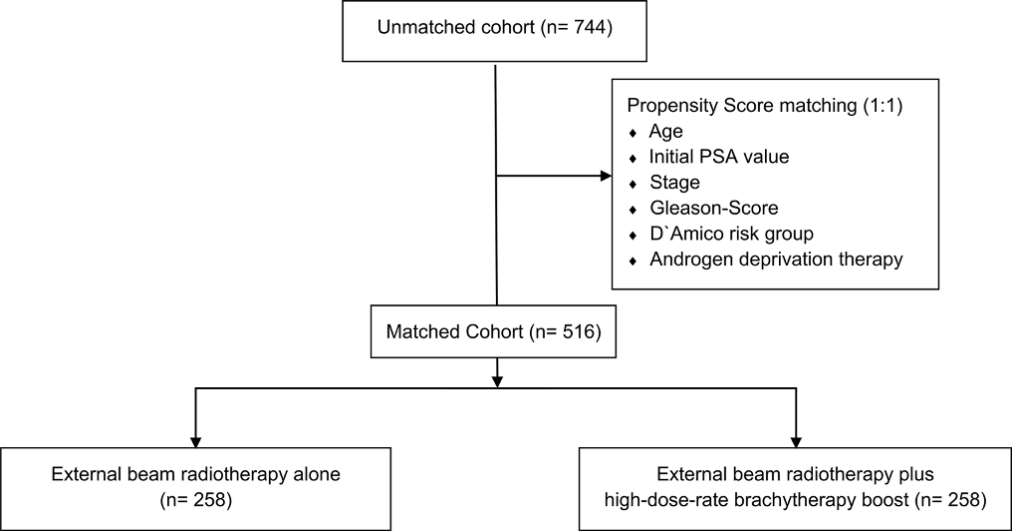
Flow diagram of the propensity score-matched analysis comparing prostate external beam radiation therapy versus external beam radiation therapy + high-dose-rate brachytherapy boost. *PSA* prostate-specific antigen

To enable independent patient matching, propensity scores were calculated for the use of EBRT alone and EBRT plus HDR-BT boost, based on logistic regression analysis. Of the 744 patients treated either with EBRT or with combined EBRT and HDR-BT boost, 258 matched pairs with a sample size of 516 patients remained after 1:1 propensity score matching. The corresponding flow diagram is presented in Fig. 1.

**Fig. 2:**
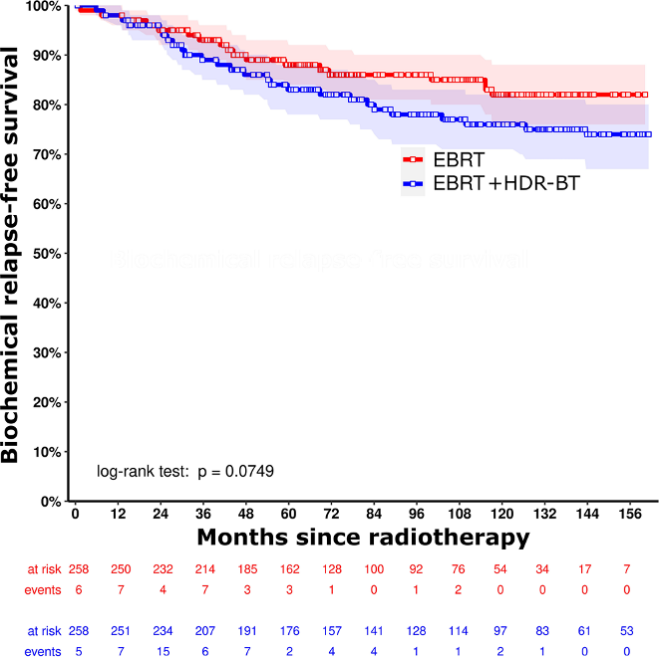
Kaplan–Meier curves for biochemical relapse-free survival for external beam radiation therapy (*EBRT*, *red line*) versus external beam radiation therapy + high-dose-rate brachytherapy boost (*EBRT* *+* *HDR-BT*, *blue line*) after propensity score matching, with corresponding 95% confidence intervals

**Fig. 3:**
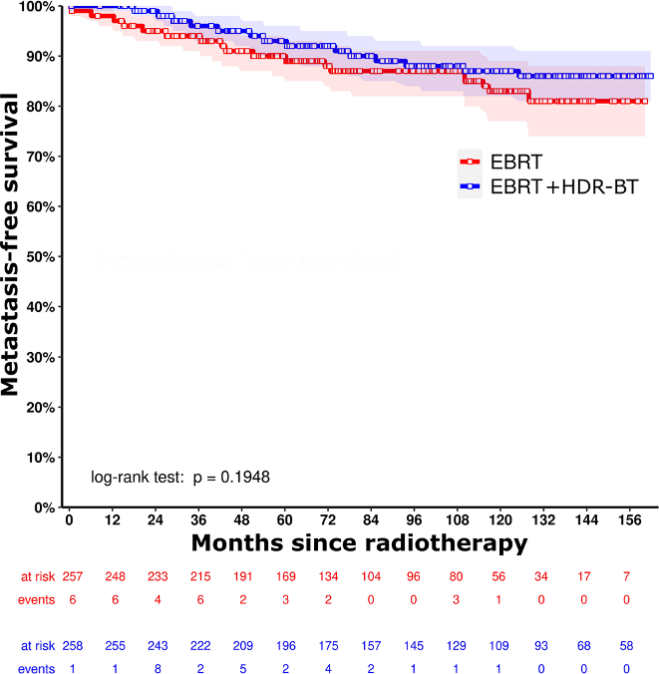
Kaplan–Meier curves for metastasis-free survival for external beam radiation therapy (*EBRT*, *red line*) versus external beam radiation therapy + high-dose-rate brachytherapy boost (*EBRT* *+* *HDR-BT*, *blue line*) after propensity score matching, with corresponding 95% confidence intervals

**Fig. 4:**
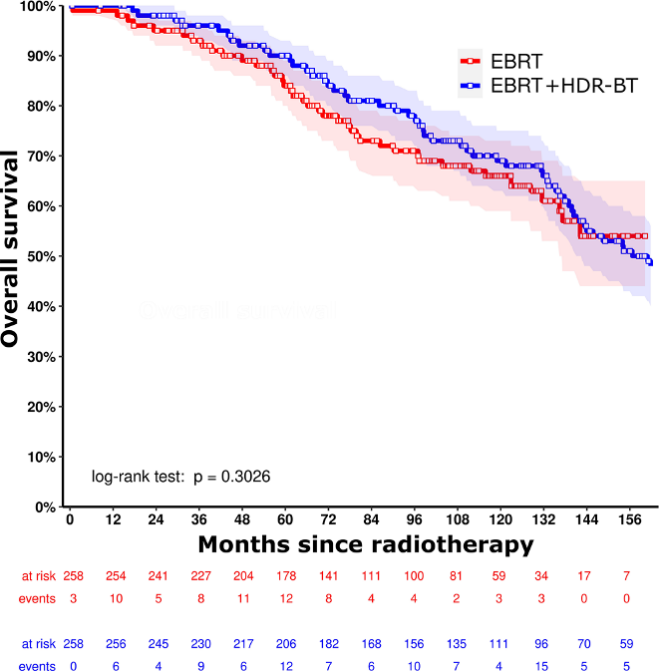
Kaplan–Meier curves for overall survival for external beam radiation therapy (*EBRT*, *red line*) versus external beam radiation therapy + high-dose-rate brachytherapy boost (*EBRT* *+* *HDR-BT*, *blue line*) after propensity score matching, with corresponding 95% confidence intervals

For the matched cohort, median follow-up (interquartile range) was 95.3 months (59.2–132.4 months) in total. In the matched cohort (*n* = 516), the estimated 10-year bRFS was 82.0% (95% CI 76.0–88.4%) versus 76.4% (95% CI 70.8–82.3%) for EBRT versus EBRT + HDR-BT boost (*p* = 0.075), respectively (Fig. 2). The estimated 10-year MFS was 82.9% (95% CI 77.0–89.2%) versus 87.0% (95% CI 82.5–91.8%) with *p* = 0.195 and the estimated 10-year OS was 65.7% (95% CI 59.0–73.2%) versus 68.9% (95% CI 62.9–75.5%) with *p* = 0.303 (Figs. 3 and 4).

In the high-risk group (*n* = 290), bRFS was worse for EBRT + HDR-BT boost: the estimated 10-year bRFS was 76.3% (95% CI 67.1–86.8%) and 66.8% (95% CI 58.8–75.9%) for EBRT alone versus EBRT + HDR-BT boost, respectively (*p* = 0.039). No differences in bRFS between the two treatments for the intermediate-risk group were observable. MFS and OS were not significantly different between the two treatments for all risk groups. Positron-emission tomography computed tomography (PET-CT) imaging follow-up was distributed equally (Table 1).

The matched cohort was investigated by multivariable Cox proportional hazard model analysis for factors associated with oncological outcome. A higher Gleason score was prognostic for lower bRFS, MFS, and OS in multivariable analysis. Higher patient age at the start of radiation therapy was prognostic for decreased MFS. The treatment group was not a prognostic factor for bRFS, MFS, and OS in multivariate analysis. The results of the Cox proportional hazard model analysis are summarized in Table 2.

**Table 2:** Multivariable Cox proportional hazard model analysis of the propensity score-matched cohort

Variable		BRFS (*N* = 514)			MFS (*N* = 512)			OS (*N* = 514)		
	*N*	HR	95% CI	*p*-value	HR	95% CI	*p*-value	HR	95% CI	*p*-value
*Age at RT*	516	0.98	0.95, 1.01	0.237	0.96	0.92, 0.99	0.007	1.02	0.99, 1.04	0.177
*PSA at diagnosis*	516	1.00	1.00, 1.01	0.079	1.00	1.00, 1.01	0.662	1.00	0.99, 1.00	0.648
*Gleason score*										
≤ 6	115	Ref	–	–	Ref	–	–	Ref	–	–
7	259	2.49	1.09, 5.68	0.031	3.20	0.94, 10.86	0.062	1.36	0.86, 2.15	0.189
≥ 8	140	4.97	2.15, 11.47	< 0.01	8.50	2.52, 28.63	< 0.01	2.15	1.30, 3.56	0.003
*Stage*										
≤ T2a	339	Ref	–	–	Ref	–	–	Ref	–	–
T2b	17	1.05	0.32, 3.44	0.936	1.97	0.58, 6.64	0.276	0.84	0.41, 1.75	0.647
≥ T2c	158	1.39	0.85, 2.27	0.192	1.75	0.95, 3.20	0.072	0.95	0.64, 1.41	0.801
*D’Amico risk class*										
Intermediate	189	Ref	–	–	Ref	–	–	Ref	–	–
High	289	1.92	0.93, 3.94	0.077	1.42	0.55, 3.66	0.469	1.25	0.75, 2.07	0.391
*ADT*										
No	242	Ref	–	–	Ref	–	–	Ref	–	–
Yes	272	0.66	0.41, 1.07	0.088	0.88	0.48, 1.59	0.665	0.86	0.62, 1.20	0.376
*Treatment*										
EBRT	257	Ref	–	–	Ref	–	–	Ref	–	–
EBRT + HDR-BT	257	1.48	0.94, 2.32	0.089	0.61	0.36, 1.04	0.068	0.89	0.64, 1.24	0.491

*ADT* androgen deprivation therapy, *bRFS* biochemical relapse-free survival, *CI* confidence interval, *EBRT* external beam radiation therapy, *HDR-BT* high-dose-rate brachytherapy, *HR* hazard ratio, *MFS* metastasis-free survival, *NA* not applicable, *OS* overall survival, *PSA* prostate-specific antigen, *Ref* reference, *RT* radiation therapy

Compared to combined EBRT + HDR-BT boost, EBRT showed significantly higher acute toxicities and higher cumulative 5‑year late GI toxicity ≥ grade 2. Acute GU toxicity ≥ grade 2 occurred in 25.9% versus 11.8% (*p* < 0.001) and acute GI toxicity ≥ grade 2 in 11.8% versus 2.7% (*p* < 0.001), respectively. Cumulative 5‑year late GU ≥ grade 2 toxicities were seen in 23.6% versus 19.2% (*p* = 0.086) and 5‑year late GI ≥ grade 2 toxicities were observed in 11.1% versus 5.0% of the patients (*p* = 0.002), respectively. Cumulative 5‑year late grade 3 GU toxicity occurred in 4.2% and 3.6% (*p* = 0.401) and GI toxicity in 1.0% and 0.3% (*p* = 0.249) in the EBRT alone versus combined treatment groups, respectively.

## Discussion

Our study demonstrated excellent 10-year bRFS, MFS, and OS for both EBRT alone and combined EBRT + HDR-BT boost. The subgroup of high-risk prostate cancer patients showed increased bRFS for dose-escalated EBRT alone. This is in contrast to the literature: regarding the comparison of conventional fractionated EBRT and EBRT + HDR-BT boost, several studies reported an improvement in bRFS for the addition of HDR-BT boost to EBRT [7, 13–16]. In the 12-year update of a randomized controlled trial, Hoskin et al. reported a significant improvement in time to relapse in the HDR-BT boost arm compared to EBRT alone (median time to relapse 137 versus 82 months) [7]. There are several possible explanations for this discrepancy: Firstly, underdosing in the seminal vesicles cannot be excluded, since the seminal vesicles were not primarily within the HDR-BT boost target volume. Additionally, multiparametric MRI was not performed regularly for HDR-BT boost treatment planning, as this imaging method became an institutional standard only during the study period (Table 1). Therefore, disease extension into the seminal vesicles may have been undetected and undertreated in a subset of the patients [17]. Secondly, several comparative studies are retrospective and may suffer from underlying imbalances in patient and tumor characteristics, which makes a direct comparison of results challenging. Compensation of differences in baseline patient and tumor characteristics by propensity score matching is a strength of our study. Moreover, in several older trials, the EBRT-alone arm used prescription doses lower than 74 Gy (EQD2). This may have influenced the outcome and conclusions of those studies [7, 18, 19]. For example, the randomized trial by Hoskin et al. used a dose of 55 Gy in 20 fractions for the EBRT arm, which, for an α/β value of 1.5 Gy, equals an EQD2 of 66.8 Gy. Our EBRT group received an EQD2 of about 83 Gy and the HDR-BT boost group an EQD2 of 100 Gy (α/β 1.5 Gy), while the experimental arm in the Hoskin trial received an EQD2 of 92 Gy [7]. Despite higher EQD2, and in contrast to the Hoskin et al. trial, we did not observe a benefit of combined EBRT + HDR-BT boost over EBRT alone. The question arises of whether an HDR-BT boost is superior compared to dose-escalated EBRT above 80 Gy.

Regarding the validity of the linear quadratic (LQ) model for dose escalation above 80 Gy, the meta-analysis by Vogelius et al. highlighted an inconsistency of the existing data in light of the results of the HYPO-RT-PC trial. Widmark et al. reported an estimated α/β ratio of 3 Gy instead of 1.5 Gy as in the historical work of Brenner and Hall, which lead Vogelius et al. to suggest a non-constant α/β ratio and/or a saturation of biochemical control when 80 Gy EQD2 is exceeded [9, 20, 21]. The increased biochemical control reported by the FLAME trial shifted the likely hypotheses toward a decreasing relative biological effect with increasing fraction size when moving beyond moderate hypofractionation [22, 23]. Moreover, Vogelius et al. discussed the presence of a time factor of an estimated 0.31 Gy/d (95% CI 0.20–0.42) [25]. In our study, the overall treatment time (OTT) differed between the two groups, with about 6.5 weeks for EBRT only versus 9 weeks for EBRT + HDR-BT boost. As this difference could not be adjusted for by propensity score matching, an influence of OTT on outcome cannot be excluded. The applicability of the LQ model at high doses per fraction and the question of optimal dose and fractionation remains to be further investigated in randomized trials [9, 24].

Morton et al. and Greco et al. recently demonstrated the importance of fractionation. Morton et al. showed that single-fraction HDR-BT was inferior to two-fraction HDR-BT [26]. For dose-escalated prostate stereotactic body radiation therapy (SBRT) up to an EQD2 of 130 Gy, Greco et al. reported a 4-year bRFS of 75.0% versus 64.0% (HR 0.76; 90% CI 0.17–3.31) for SBRT with 5 × 9 Gy versus 1 × 24 Gy for unfavorable disease, respectively [27]. Two findings are of interest: firstly, one-fraction dose escalation appears to be inferior to dose escalation in more fractions. Secondly, the bRFS in the study of Greco et al. is lower than in a meta-analysis of over 6000 patients from Jackson et al., in which SBRT demonstrated a 5-year bRFS of 95.3% (95% CI 91.3–97.5%). Notably, increasing dose was significantly associated with improved bRFS, but only 38% of all studies included patients with high-risk disease and most studies did not report bRFS for high-risk disease separately [28].

Dose escalation above 80 Gy in unfavorable prostate cancer is supported by the results of the FLAME trial by Kerkmeijer et al.: dose escalation to 95 Gy for the dominant intraprostatic lesion improved 5‑year bRFS up to 92% in a cohort with 84% high-risk patients [22]. For brachytherapy, the ASCENDE-RT trial impressively demonstrated improved bRFS with appliance of low-dose-rate brachytherapy boost over EBRT with 78 Gy, which supports brachytherapy as valid dose-escalation strategy [29]. To summarize, promising data for dose escalation in localized prostate cancer exists, but further randomized controlled trials are necessary to elucidate the interplay between biochemical control, the LQ model, and prescription of high doses per fraction for dose escalation above 80 Gy.

Besides benefits in bRFS, it is crucial to elucidate whether dose escalation influences clinically meaningful endpoints (for example, MFS and OS). Notably, MFS and OS were not significantly different for the two treatment modalities in the matched cohort of our study. In addition, dose escalation by EBRT is often limited by increased toxicity. In this context, dose escalation by HDR-BT has a favorable toxicity profile: in our study, late severe toxicities were not significantly different between the treatment cohorts. The combined treatment showed decreased acute toxicities grade ≥ 2 as well as lower moderate gastrointestinal toxicity compared to EBRT. Combined with excellent MFS and OS, EBRT + HDR-BT boost represents a competitive treatment regimen for intermediate- and high-risk prostate cancer.

Our study has limitations. Despite extensive propensity score matching, undetected differences in baseline characteristics between the treatment groups may have remained. Notably, we did not account for comorbidities and patients in the EBRT + HDR-BT group were, despite matching, younger, which could have influenced the outcome, especially overall survival. Differences in MRI staging may have led to a skewed stage distribution (Table 1): in the matched cohort, 154 out of 258 cases (59.7%) received MRI before treatment in the EBRT-only group, whereas only 49 out of 258 cases (19.0%) had MRI in the EBRT + HDR-BT group (*p* = < 0.001). Moreover, patients with known seminal vesicle involvement may have been preferably treated with EBRT alone, possibly resulting in a negative selection of the EBRT group.

## Conclusion

Dose-escalated EBRT with an EQD2 above 80 Gy and combined EBRT + HDR-BT boost showed no significant differences in outcome in a large propensity score-matched cohort. EBRT + HDR-BT boost demonstrated a trend to worse biochemical relapse-free survival compared to EBRT alone for the subgroup of high-risk prostate cancers. The combined treatment provided a favorable toxicity profile with lower acute toxicity and late gastrointestinal toxicity ≥ grade 2. EBRT as well as EBRT + HDR-BT boost are both excellent options for dose-escalating radiotherapy above 80 Gy (EQD2) in localized prostate cancer.
